# Deep cryo treated tungsten carbide tools on AISI 1045 steel turning through grey relational analysis and preference selection index

**DOI:** 10.1038/s41598-025-02263-w

**Published:** 2025-05-27

**Authors:** P Raja, M Sakthivel, T Satish Kumar, Jana Petrů, Kanak Kalita

**Affiliations:** 1 Department of Mechanical Engineering, Prathyusha Engineering College, Chennai, 602025 India; 2 Department of Mechanical Engineering, Adhiyamaan College of Engineering, Hosur, 635109 India; 3 Department of Mechanical Engineering, Amrita School of Engineering, Amrita Vishwa Vidyapeetham, Coimbatore, 641112 India; 4 Department of Machining, Assembly and Engineering Metrology, Faculty of Mechanical Engineering, VSB-Technical University of Ostrava, Ostrava, 70800 Czech Republic; 5 Department of Mechanical Engineering, Vel Tech Rangarajan Dr. Sagunthala R&D Institute of Science and Technology, Avadi, 600 062 India

**Keywords:** Cryo treatment, Turning, Taguchi technique, Grey relation analysis, Preference selection index, Surface roughness, Tool wear, Engineering, Mechanical engineering

## Abstract

Global competition and increasing environmental concerns have compelled manufacturing industries to reduce energy consumption and enhance product quality. This, in turn, helps increase the production rate. In this context, the machining performance is largely influenced by the selection of process parameters and the condition of the cutting tool. The present study is based on an experiment involving the use of an uncoated, deep cryogenically treated tungsten carbide tool for machining AISI 1045 steel. The outcomes were evaluated using Grey Relational Analysis (GRA) and the Preference Selection Index (PSI). Both ANOVA methods indicated that feed rate, cutting speed, the use of deep cryo-treated tools, and depth of cut had the most significant effects. The optimal parameter settings identified include a deep cryo-treated tool, a cutting speed of 120 m/min, a feed rate of 0.05 mm/rev, and a depth of cut of 1.00 mm. This approach demonstrated that the feed rate had the greatest influence on flank wear and surface roughness, both of which were also significantly affected by cutting speed and depth of cut. Moreover, the deep cryo-treated tool outperformed the untreated tool, resulting in reductions in surface roughness and flank wear by 17% and 7%, respectively. Deep Cryogenic Treatment (DCT) has thus shown promise in enhancing the performance of tungsten carbide cutting tools used in machining operations. This study specifically investigated the effect of DCT on tool wear and surface finish during the turning of AISI 1045 steel.

## Introduction

Machining processes and energy conservation have become essential strategies in many countries as the global energy crisis intensifies. The primary objective of all industrial activities, particularly in manufacturing, is to reduce energy consumption. To achieve this, optimization methodologies are implemented to evaluate process parameters and minimize energy usage during machining operations. The fundamental machining process of removing material from the outer surface of a rotating component involves the use of a cutting tool. Therefore, the careful selection of a tool—based on both quality and cost—is crucial to the machining process. As a result, a key focus of contemporary machining research is to enhance performance through the implementation of advanced techniques. One such technique that complements conventional thermal treatment is the cryogenic treatment of cutting tools. Deep cryogenic treatment (DCT) is performed at extremely low temperatures, typically ranging from − 140 °C to −196 °C^1^. Tools coated via cryo-physical vapor deposition have shown a significant effect on the rotary machining capability of nickel-based Hastelloy C22. In one study, DCT improved surface roughness ($\:{R}_{a}$) and reduced cutting forces by 99.5% and 19.7%, respectively, compared to untreated tools (UT)^2^. Further experiments have been conducted on tungsten carbide insert tools at two temperature levels: −110 °C and − 196 °C. These findings demonstrate that cryo-treated tools offer superior performance^3,4^. Additionally, tools subjected to DCT have shown a notable increase in tool life when compared to those treated with shallow cryogenic treatment (SCT)^5–8^.

A study evaluated the responses of attrition, chip morphology, vibrations, cutting force, and surface roughness during machining. The findings revealed that cryo-treated inserts exhibited reduced cutting force, vibration, and tool attrition^9^. DCT-coated TiN tungsten carbide and TiCN tools were also employed for machining medium-carbon steel. The results showed that as cutting speed increased, both cutting force and surface roughness ($\:{R}_{a}$) also increased. The microstructural analysis of the DCT tools revealed a thin and well-refined carbide structure, indicating enhanced wear resistance in cementite-based tungsten carbide tools. Cobalt became denser and contained a greater number of carbide particles due to these substantial modifications^10^. Cryo-treatment significantly improved the abrasion resistance of the tools, achieving optimal wear resistance and surface finish after 24 h of DCT. Furthermore, DCT significantly enhanced tool hardness, with the 24-hour treated tool showing the highest improvement (10.87%). An analysis of variance (ANOVA) and optimization of cryo-treatment types indicated that cutting speed had the most substantial effect on flank wear, while feed rate most significantly influenced surface roughness ($\:{R}_{a}$)^11^. Another study examined the corrosion behavior of three distinct grades of high-speed steel (HSS), utilizing a heat treatment process that included DCT. The results showed a 31% enhancement in both hardness and microstructure of the steel^12^. Kamalakannan et al.^13^ investigated the effect of deep cryogenic treatment (DCT) on the performance of cutting tools during the turning of AISI 1045 steel. Various studies have explored the impact of DCT on high-speed steel (HSS), untreated carbide (UTC), conventionally treated carbide (CTC), and uncoated deep cryo-treated (DCT) tools. Grey Relational Analysis (GRA) has been widely employed to optimize machining parameters by assessing factors such as flank wear, surface roughness, cutting force, and temperature. The findings consistently demonstrate that DCT tools outperform untreated tools, exhibiting reduced cutting forces, lower temperatures, decreased tool wear, and improved surface finish. Statistical tools such as Minitab and Design of Experiments (DOE) have further validated these results, reinforcing the advantages of DCT in enhancing tool life and machining performance. Simranpreet et al.^4^ examined the role of cryogenic treatment in enhancing the performance and tool life of tungsten carbide inserts. While previous studies have primarily focused on quantifying the percentage improvement in tool life, limited attention has been given to understanding the underlying mechanisms. Research indicates that deep cryogenic treatment (DCT) at − 196 °C significantly enhances wear resistance and machining performance compared to shallow cryogenic treatment (SCT) at − 110 °C. Key evaluation parameters such as flank wear and surface roughness demonstrate that DCT-treated inserts outperform both untreated and SCT-treated inserts. These findings underscore the effectiveness of DCT in extending tool life and improving machining efficiency in turning operations. Some researchers^14^ aimed to determine whether reaming operations influenced various environmental characteristics, employing the L18 Taguchi orthogonal array (OA) and Grey Relational Analysis (GRA) to evaluate cutting speed and feed rate. The optimal results of the Grey Relational Grade (GRG) demonstrated that GRA was highly effective in resolving multi-response problems during the reaming process^15^. Some researchers also examined the effects of standard and cryogenic treatments on high-carbon and chromium-alloy steels. Six input parameters were considered in the experiment, with output responses including cutting rate, material removal rate (MRR), and surface finish. Their results show that combining GRA with the Taguchi technique led to satisfactory performance outcomes^16^. Numerous studies have successfully applied the Preference Selection Index (PSI), a simple multi-objective optimization method, to simultaneously minimize surface roughness ($\:{R}_{a}$) and maximize MRR by selecting optimal cutting conditions^17^. One such study used a high-speed steel (HSS) tool to mill 060 A4 steel, employing the Taguchi method to design an experimental matrix and using PSI for multi-objective optimization^18^. Decision-making involving multiple turning parameters has led to the development of new multi-criteria decision-making approaches^19^. Many researchers have initially used single-response optimization techniques to identify the best parameter settings; however, these methods often fall short due to operational complexity and limited scope.

To address these challenges and resolve ambiguity in machining processes, experiments were conducted using two optimization techniques— GRA and PSI. These methods demonstrate a high level of effectiveness in selecting appropriate process parameters, proving valuable for both researchers and industrial applications.

## **Material and methodology**

Table 1 illustrates the work piece’s material composition^5^. Automobile shafts and axles frequently employ AISI 1045 steel^20^. The tool was uncoated tungsten carbide, which has been utilized in industry to date^21^. Its specifications were SNMG 120,408 ML TTR with a chip breaker.

**Table 1:** Material composition of AISI 1045 steel.

Elements	C	Si	Cr	V	W	Mo
Weight (%)	0.974	0.115	3.955	1.891	6.509	4.95

### Cryo treatment

**Fig. 1:**
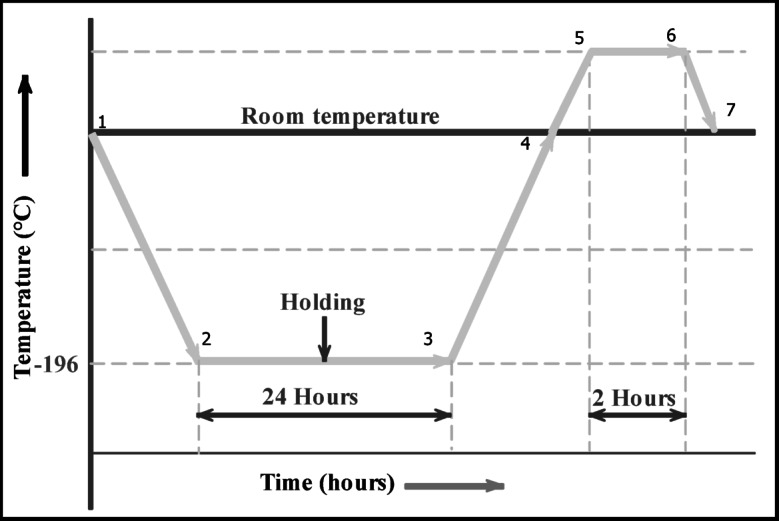
Cryo treatment.

Cryo treatment, a sub-zero thermal treatment, is an ecologically responsible substitute to conventional techniques that enhances wear resistance, tool life, dimensional reliability, and component quality. Figure 1 schematically illustrates the cryo-treatment of the instrument with single-tempering cycles. The cooling cycle (CC) required the use of a computerized temperature controller to regulate the temperature at a rate of 0.5 to 1 min and lasted for 8 h (1–2). During the soaking process, stations 2 to 3 maintained a DCT temperature of −196 °C for 86,400 s. The chamber temperature reached its maximum eight hours after the warming temperature was set (3–4). Once CC was complete, tempered the material at 200 °C for 7600 s (4–5) maintained the material at that temperature for two hours (5–6) to alleviate tension, and then cooled it to room temperature during the subsequent cooling series (6–7)^22,23^.

### Experimental work

**Fig. 2:**
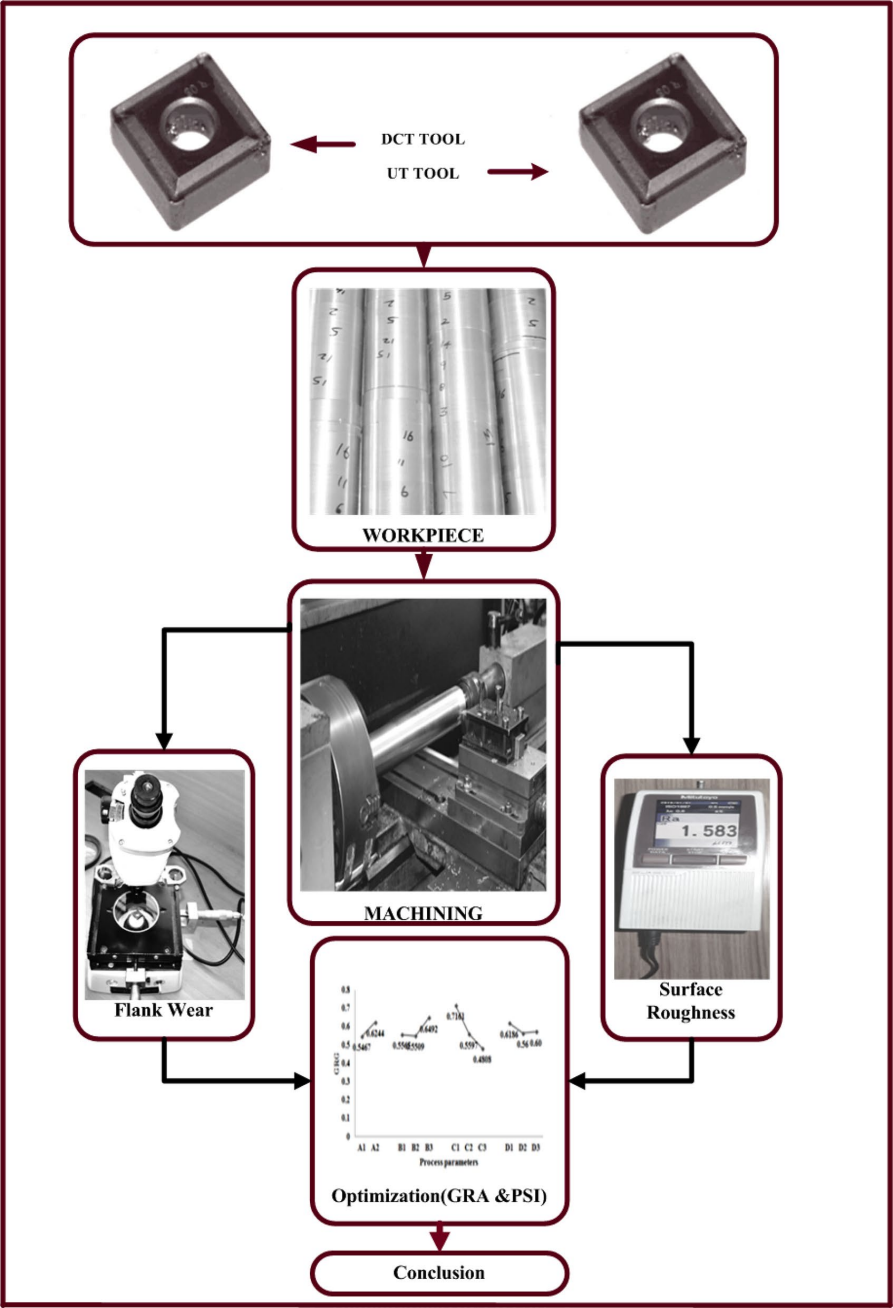
Experimental workflow of this research.

The tool manual and the literature were used to select the experiments conducted by UT and DCT tools on various process parameter levels^24^. Turning was performed on the Kirloskar lathe equipment, Turn Master 25. The experiment was conducted in accordance with the flow depicted in Fig. 2; Table 2, and the process parameters and their order were implemented during the machining process. The response of $\:{R}_{a}$values was investigated using a Mitutoyo tester; Surf test-210 parameters cutoff length for the roughness was 15 mm and a metallurgical microscope, Metzer VFM 9100. Additionally, flank wear (VB max) was investigated.

**Table 2:** Process parameter and their ranges.

Sl.No.	Description	Units	Ranges		
			1	2	3
1	Cutting speed (v)	m/min	49.95	70.05	120.00
2	Feed rate(s)	mm/rev	0.050	0.075	0.100
3	Depth of cut (t)	mm	0.100	0.750	1.000

### Optimization techniques

The two optimization techniques— GRA and PSI, were used in the research.

### Grey relation analysis (GRA)

GRA (grey relational analysis) used for resolving problems with multiple component and complicated connection. The normalization is initialized first according to the preference of higher the better or lesser the better. The deviation is analyzed and the grey relational rank and rank is determined. The grey relational analysis is executed mainly to determine the prime parametric combination. The steps required to calculate the various criteria in GRA are as follows—.

For a MCDM problem consisting of $\:m$ alternatives and $\:n$ criteria, let $\:D={x}_{ij}$ be a decision matrix, where $\:{x}_{ij}\in\:\mathbb{R}$.

1 $$\:D=\left[\begin{array}{cccc}{x}_{11}&\:{x}_{12}&\:\cdots\:&\:{x}_{1n}\\\:{x}_{21}&\:{x}_{22}&\:\cdots\:&\:{x}_{2n}\\\:\vdots&\:\vdots&\:\ddots\:&\:\vdots\\\:{x}_{m1}&\:{x}_{m2}&\:\cdots\:&\:{x}_{mn}\end{array}\right]$$

The normalization of two types of data i.e. better when higher type or better when lower is evaluated using Eq. (2) or (3) respectively. After normalization the data ranges from 0 to 1.

2 $$\:{M}_{ij}=\frac{{N}_{ij}-\text{min}\left({N}_{ij}\right)}{\text{max}\left({N}_{ij}\right)-\text{min}\left({N}_{ij}\right)}$$

3 $$\:{M}_{ij}=\frac{\text{m}\text{a}\text{x}\left({N}_{ij}\right)-{N}_{ij}}{\text{max}\left({N}_{ij}\right)-\text{m}\text{i}\text{n}\left({N}_{ij}\right)}$$

where $$\:i,\:j=\:\text{1,2},3\dots\:\dots\:n$$

4 $$\:\epsilon\:=\text{max}\text{v}\text{a}\text{l}\text{u}\text{e}\:\text{a}\text{f}\text{t}\text{e}\text{r}\:\text{n}\text{o}\text{r}\text{m}\text{a}\text{l}\text{i}\text{z}\text{a}\text{t}\text{i}\text{o}\text{n}-\text{v}\text{a}\text{l}\text{u}\text{e}\:\text{o}\text{f}\:\text{t}\text{h}\text{e}\:\text{c}\text{u}\text{r}\text{r}\text{e}\text{n}\text{t}\:\text{r}\text{o}\text{w}$$

The separate deviation sequence for each experiment was calculated using Eq. (5) with the normalized value obtained.

5 $$\:{C}_{ij}=\frac{{\varDelta\:}_{minimum}+\epsilon\:{\varDelta\:}_{maximum}}{current\:value+\epsilon\:{\varDelta\:}_{maximum}}$$

In order to obtain the grey relation coefficient Eq. (5) is used. The rank was obtained from the grey relational coefficient the for most responsive process parameter.

### Preference selection index (PSI)

Maniya and Bhatt created a PSI approach for determining multi-target optimization problems^25^, and the methodology consisted of the steps discussed below—.

The MCDM problem in terms of Eq. (1). If the response is benefit type i.e. larger values are anticipated than the normalization is done using Eq. (6)

6 $$\:{n}_{ij}=\frac{{x}_{ij}}{{{x}_{j}}^{max}}$$

If the response is cost type i.e. smaller values are anticipated than the normalization is done using Eq. (7)

7 $$\:{n}_{ij}=\frac{{{x}_{j}}^{min}}{{x}_{ij}}$$

Mean value of each normalized value of each response is calculated as

8 $$\:\text{N}=\frac{\sum\:_{i=1}^{n}{n}_{ij}}{n}$$

Next a preference variation value among each response is calculated as

9 $$\:{\varphi\:}_{j}=\sum\:_{i=1}^{n}{\left[{n}_{ij}-n\right]}^{2}$$

Variation in the preference value for each response is calculated as

10 $$\:{{\Omega\:}}_{j}=\left[1-{\varphi\:}_{j}\right]$$

Then the overall preference value is obtained for individual response by

11 $$\:{\omega\:}_{j}=\frac{{{\Omega\:}}_{j}}{\sum\:_{j=1}^{m}{{\Omega\:}}_{j}}\:\text{s}\text{u}\text{c}\text{h}\:\text{t}\text{h}\text{a}\text{t}\:\sum\:_{j=1}^{m}{\omega\:}_{j}=1$$

To calculate the PSI values of the alternatives with the help of Eq. (12).

12 $$\:{\eta\:}_{i}=\sum\:_{j=1}^{n}{y}_{ij}{w}_{j}$$

## Results and discussion

Under dry conditions, the experiment involved UT and DCT tungsten carbide tools with different parameters. In this results section, experimental and multi-objective optimization values are discussed. Table 3 shows how experimental values were determined during the machining of the measured response values.

**Table 3:** Measured experimental values.

Exp.	v (m/min)	s (mm/rev)	t (mm)	UT		DCT	
				$\:{R}_{a}$ (µm)	VB max (mm)	$\:{R}_{a}$ (µm)	VB max (mm)
1	49.95	0.050	0.10	4.546	0.020**(Low)**	4.030	0.015**(Low)**
2	49.95	0.075	0.75	4.677	0.035	4.630	0.032
3	49.95	0.100	1.00	4.947**(High)**	0.051	4.946**(High)**	0.05
4	70.05	0.050	0.75	3.294	0.04	2.839	0.028
5	70.05	0.075	1.00	3.869	0.04	3.466	0.034
6	70.05	0.100	0.10	3.874	0.062	3.754	0.035
7	120	0.050	1.00	2.033**(Low)**	0.041	1.933**(Low)**	0.04
8	120	0.075	0.10	2.340	0.06	2.246	0.052
9	120	0.100	0.75	2.630	0.075**(High)**	2.251	0.065**(High)**

### Experimental results

**Fig. 3:**
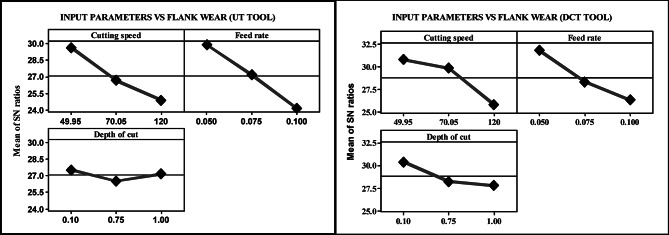
Response graph of VB max for UT and DCT tungsten carbide tools.

**Fig. 4:**
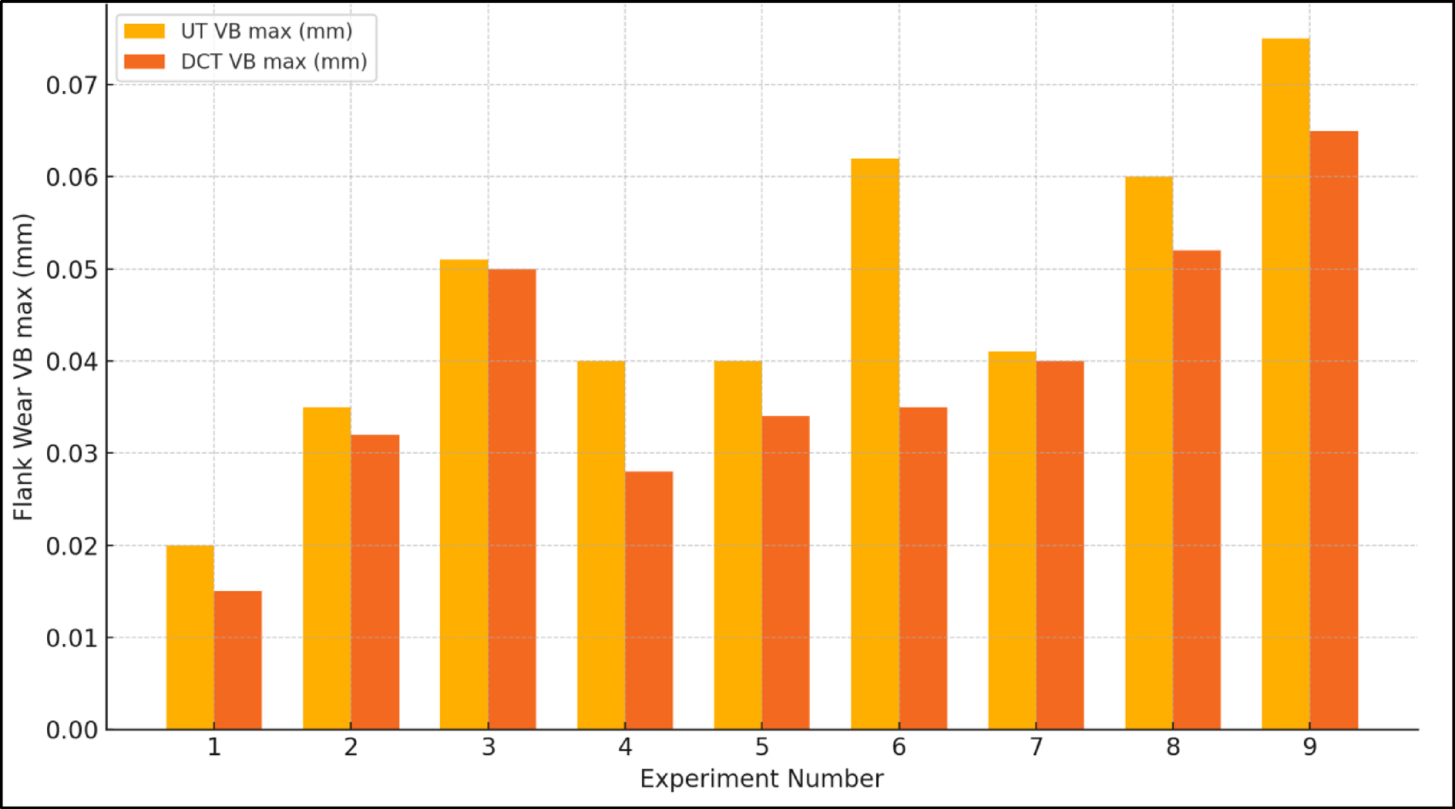
Comparison of UT and DCT tungsten carbide tools on flank wear.

Table 3 suggests that the lowest cutting speed, feed rate, and depth of cut resulted in minimal flank wear. The UT and DCT tools had values of 0.020 mm and 0.015 mm, respectively. The most significant response was observed as the feed rate increased, as well as the depth of cut and cutting speed. The UT and DCT tools had values of 0.075 mm and 0.065 mm, in that order. DCT attained levels of 13% and 25%, respectively. Figure 3 depicts the response diagram for VB max. Both showed a consistent trajectory towards reducing and increasing flank wear. The VB max was the lowest must reduce the cutting speed, feed rate, and depth of cut. The levels were as follows: a feed rate of 0.05 mm per rev, a depth of cut of 0.10 mm, and a lower cutting speed of 318 rpm. Typically, the cutting speed increases in tandem with the feed rate and depth of cut, leading to an increase in the maximum VB. The blunting of the leading enhances the force, which in turn increases the wear, as an impact of the increased friction among the blunted tool edge and the workpiece^22^. Nevertheless, the DCT tool demonstrated a 11% reduction in cutting force when compared to the UT tool in machining^26^. The DCT is superior to the UT tool under identical cutting conditions, as illustrated in Fig. 4.

**Fig. 5:**
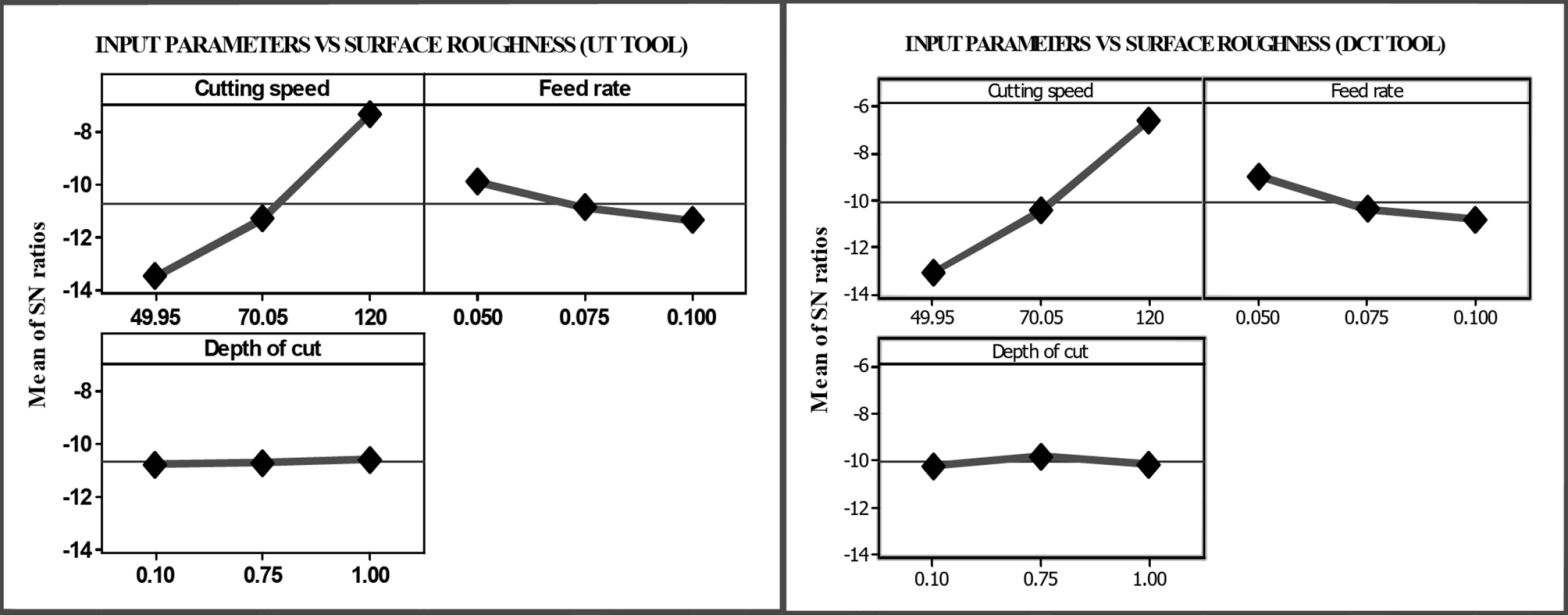
Response graph of $\:{R}_{a}$ for UT and DCT tungsten carbide tools.

**Fig. 6:**
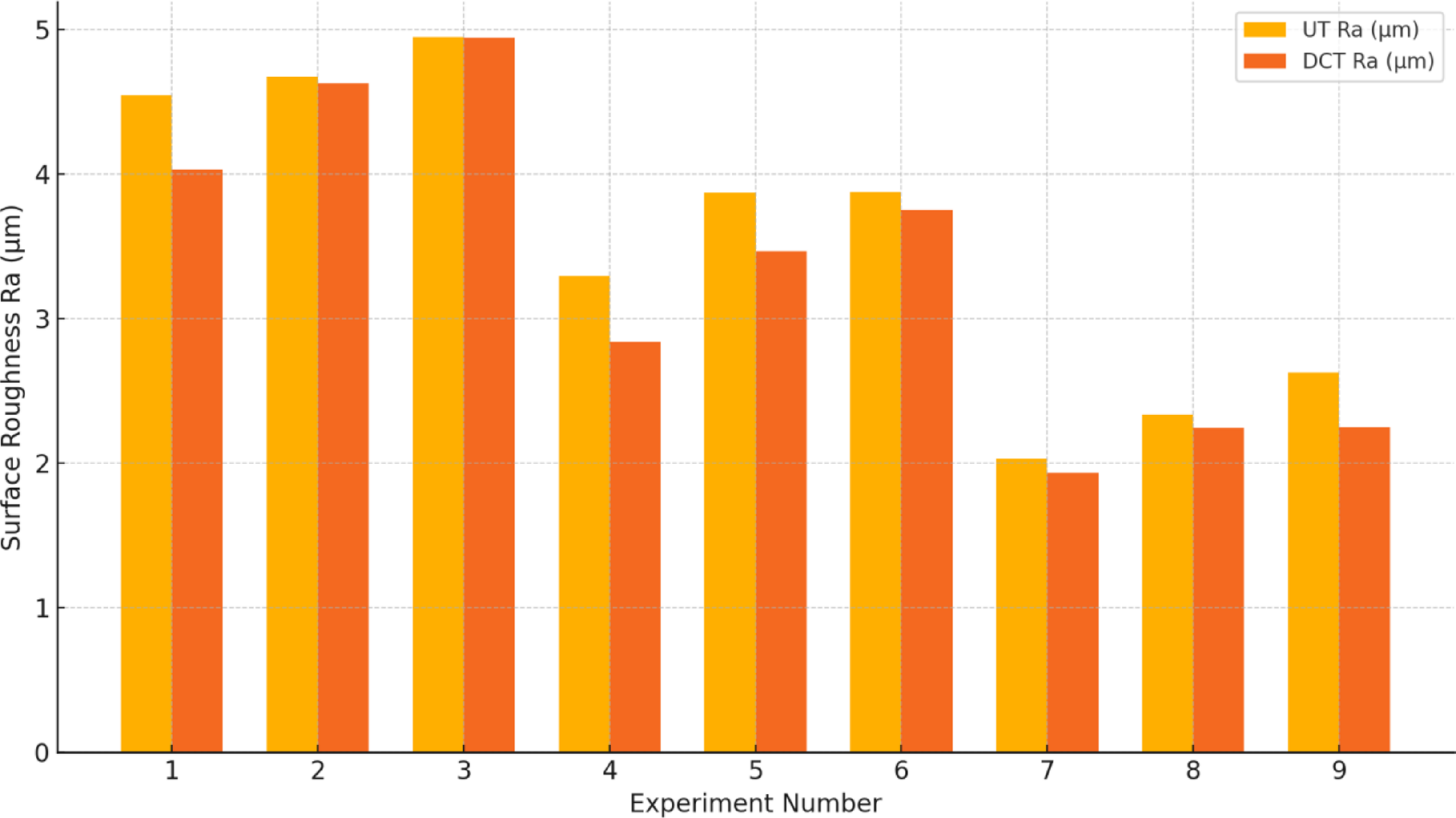
Comparison of UT and DCT tungsten carbide tools on surface roughness.

The lowest $\:{R}_{a}$ value is attained at a higher cutting speed and a lower feed rate, as illustrated in Table 3. In the case of the UT and DCT tools, the values were 4.947 and 4.946 μm, respectively. Lower cutting velocities, higher feed rates, and a greater depth of cut elicited the most significant response. The UT and DCT instruments had $\:{R}_{a}\:$values of 2.033 μm and 1.933 μm, respectively. The DCT tool obtained a higher level of 0.02% and a reduced level of 5%. Figure 5 displays the $\:{R}_{a}$ response graphs for the two tools, which correspondingly followed the same trend to attain lower and higher $\:{R}_{a}$ values. This suggests that the cutting speed is increased by increasing the feed rate and depth of cut in order to identify an exceptional $\:{R}_{a}$ in machining. The feed rate was set to 0.050 mm/rev, the cutting speed was 120 m/min rpm, and the depth of cut was 0.10 mm. The cutting speed increased as a result of the continuous reduction in built-up edge formation, and $\:{R}_{a}$ decreased. In general, the $\:{R}_{a}$ is increased by a higher input rate and a deeper cut. The DCT and UT tools’ performances are illustrated in Fig. 6. Nevertheless, the microstructure of the rigid and flexible phases is altered by DCT, which results in the precipitation of phase carbides to prolong the life of the inserts. This process reduces the $\:{R}_{a}\:$during milling. Nevertheless, the DCT tool demonstrated a 7% reduction in $\:{R}_{a}$ compared to the UT tool during machining

### GRA results

**Table 4:** Grey relation analysis results.

Exp.	Tools	Raw values		Normalization		Deviation sequence		Grey coefficient		GRG
		VB max (mm)	$\:{R}_{a}$(µm)	VB max (mm)	$\:{R}_{a}$(µm)	VB max (mm)	$\:{R}_{a}$(µm)	VB max (mm)	$\:{R}_{a}$(µm)	
1	UT tool	0.02	4.546	1.000	0.138	0.000	0.862	1.000	0.367	0.710
2		0.035	4.677	0.733	0.093	0.267	0.907	0.652	0.355	0.525
3		0.051	4.947	0.333	0.000	0.667	1.000	0.429	0.333	0.413
4		0.04	3.294	0.556	0.567	0.444	0.433	0.529	0.536	0.507
5		0.04	3.869	0.689	0.370	0.311	0.630	0.616	0.442	0.490
6		0.062	3.874	0.067	0.368	0.933	0.632	0.349	0.442	0.416
7		0.041	2.033	0.556	1.000	0.444	0.000	0.529	1.000	0.694
8		0.06	2.34	0.111	0.895	0.889	0.105	0.360	0.826	0.633
9		0.075	2.63	−0.222	0.795	1.222	0.205	0.290	0.709	0.501
10	DCT tool	0.015	4.03	1.111	0.315	−0.111	0.685	1.286	0.422	0.715
11		0.032	4.63	0.667	0.109	0.333	0.891	0.600	0.359	0.506
12		0.05	4.946	0.333	0.000	0.667	1.000	0.429	0.333	0.440
13		0.028	2.839	0.822	0.723	0.178	0.277	0.738	0.644	0.625
14		0.034	3.466	0.556	0.508	0.444	0.492	0.529	0.504	0.522
15		0.035	3.754	0.667	0.409	0.333	0.591	0.600	0.458	0.534
16		0.04	1.933	0.556	1.034	0.444	−0.034	0.529	1.074	0.749*
17		0.052	2.246	0.289	0.927	0.711	0.073	0.413	0.872	0.647
18		0.065	2.251	0.000	0.925	1.000	0.075	0.333	0.870	0.584

**Fig. 7:**
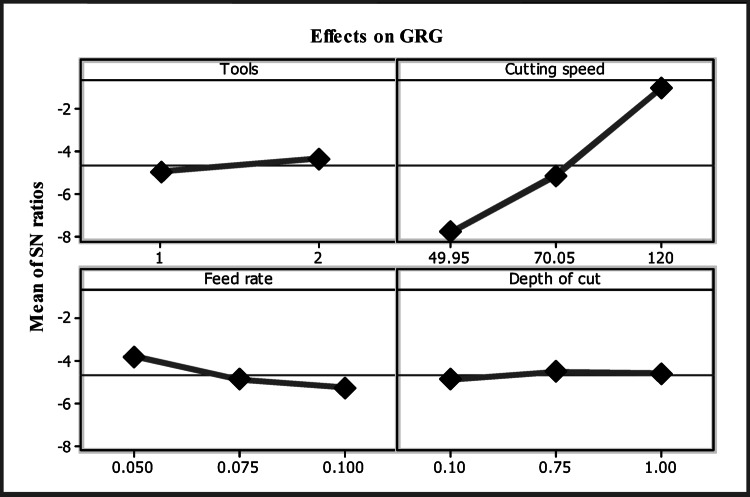
Influences on GRG.

Response and experimental values are presented in Table 4. Where there are numerous optimization targets, the Taguchi strategy with the GRA performs better. Identification of relevant quality qualities can reduce a multi-objective functional problem to a single-objective problem^27^. The values at level two for tools, level three for cutting speed, level one for feed rate and level one for depth of cut indicate superior performance in the main effect plots in Fig. 7.

Table 5 displays the highest GRG contribution rank for each variable, indicating a value at the optimal parameter level. Using the cutting speed, tools, and depth of cut, which are significant parameters with ratings of 1, 2, 3, and 4, it was found that the input rate was the most controllable in the process. In addition, the optimal response in Experiment 16 was a higher GRG, as illustrated in Table 4. Table 5 provides a list of the most optimal variables. Each tool, A2, B3, C1, and D1, has a different feed rate and depth of cut, and its cutting speed ranges from two to three. The optimal process parameters for a DCT tool (A2) were a cutting speed of 120 m/min (B3), a feed rate of 0.050 mm/rev (C1), and a depth of cut of 0.10 mm (D1).

**Table 5:** GRG analysis.

Factors	Levels 1	Levels 2	Levels 3	Max. - Min.	Rank
Tools	0.5434	0.5913*		0.0479	4
Cutting speed	0.5515	0.5155	0.635*	0.1195	2
Feed rate	0.6667*	0.5538	0.4815	0.1852	1
Depth of cut	0.6092*	0.5415	0.5514	0.0578	3

The ANOVA is employed to estimate the GRG and its effect, as seen in Table 6. The experiment’s control factors were the depth of cut, feed rate, cutting speed, and the implement. The purpose of the contribution was to alter the feed rate in the experiment. Cutting speed, tool, and depth of cut all of which have direct effects on cutting and adhesion came in next at 55%, 24%, 8%, and 5%, correspondingly tool wear is influenced by the feed rate and cutting speed^28^.

**Table 6:** ANOVA results.

Notations	Factors	DF	Sequential sums of squares	Adjusted sum of squares	Mean Sum of Squares	% of effect
A	Tools	1	0.01036	0.01036	0.010356	5
B	Cutting speed	2	0.04509	0.04509	0.022543	24
C	Feed rate	2	0.10462	0.10462	0.052311	55
D	Depth of cut	2	0.01603	0.01603	0.008015	8
	Total	17	0.19123			

### PSI results

**Table 7:** Preference selection index results.

Exp.	Tools	Raw values		Normalization		Preference variation value		Overall preference value		PSI Ranking Value
		VB max (mm)	$\:{R}_{a}$(µm)	VB max (mm)	$\:{R}_{a}$(µm)	VB max (mm)	$\:{R}_{a}$(µm)	VB max (mm)	$\:{R}_{a}$(µm)	
1	UT tool	0.02	4.546	1.000	0.425	1.000	0.048	−0.036	0.441	0.710
2		0.035	4.677	0.571	0.413	0.000	−0.32	−0.021	0.428	0.525
3		0.051	4.947	0.392	0.391	−0.023	−0.802	−0.014	0.405	0.413
4		0.04	3.294	0.500	0.587	−0.001	−0.485	−0.018	0.608	0.507
5		0.04	3.869	0.500	0.500	−0.001	−0.485	−0.018	0.518	0.490
6		0.062	3.874	0.323	0.499	−0.049	−1.046	−0.012	0.517	0.416
7		0.041	2.033	0.488	0.951	−0.003	−0.518	−0.018	0.985	0.694
8		0.06	2.34	0.333	0.826	−0.044	−1.008	−0.012	0.856	0.633
9		0.075	2.63	0.267	0.735	−0.077	−1.267	−0.010	0.761	0.501
10	DCT tool	0.015	4.03	1.333	0.480	0.622	0.693	−0.048	0.497	0.715
11		0.032	4.63	0.625	0.417	0.006	−0.219	−0.022	0.433	0.506
12		0.05	4.946	0.400	0.391	−0.020	−0.776	−0.014	0.405	0.440
13		0.028	2.839	0.714	0.681	0.028	−0.092	−0.026	0.705	0.625
14		0.034	3.466	0.588	0.558	0.001	−0.286	−0.021	0.578	0.522
15		0.035	3.754	0.571	0.515	0.001	−0.32	−0.021	0.533	0.534
16		0.04	1.933	0.500	1.000	−0.001	−0.485	−0.018	1.036	0.749*
17		0.052	2.246	0.385	0.861	−0.025	−0.826	−0.014	0.892	0.647
18		0.065	2.251	0.308	0.859	−0.056	−1.104	−0.011	0.890	0.584

**Fig. 8:**
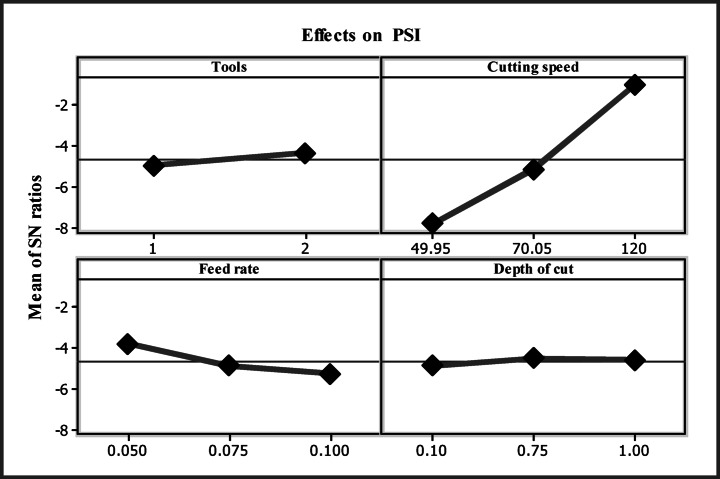
Influences on PSI.

Table 7 shows the responses that were assessed using the PSI values. The PSI approach involves choosing the best option from a list of choices in the application of the decision-making process without assessing the relative weights of various attributes^29^. The main effect diagrams in Fig. 8 demonstrate that the values at level two for instruments, level three for cutting speed, level one for feed rate, and level one for depth of cut exhibit superior performance.

Equations 6–12 determined the PSI rank of each value, and Table 7 summarizes the obtained results. The optimal response was experiment 16, same as shows in Table 7. Table 8 determines the PSI values at each level. Table 8 indicates the maximum value at the optimal parameter level.

**Table 8:** PSI results.

Factors	Levels 1	Levels 2	Levels 3	Maximum - Minimum	Rank
Tools	0.5956	0.6414 *		0.0458	3
Cutting speed	0.4087	0.5573	0.8896 *	0.4809	1
Feed rate	0.6848 *	0.5992	0.5716	0.0652	2
Depth of cut	0.5989	0.6196	0.6371*	0.0382	4

Table 8 presents the list of variables that are most appropriate. In the event that there were two tools, the cutting speed was three, the feed rate was one, and the depth of cut of each of the three tools was A2 B3 C1 D3. The most effective methods were the DCT tool (A2), a cutting speed of 120 m/min (B3), a feed rate of 0.050 mm/rev (C1), and a depth of cut of 1.0 mm (D3). Table 9 illustrates the estimation of PSI and its influence using ANOVA. The cutting speed and feed rate of the experiment were found to be strongly influenced by the contribution, followed by the tool and depth of cut, which were 91%, 5%, 1%, and 1%, respectively.

**Table 9:** ANOVA results.

Notations	Factors	DF	Sequential sums of squares	Adjusted sum of squares	Mean Sum of Squares	% of effect
A	Tools	1	0.009437	0.009437	0.009437	1
B	Cutting speed	2	0.727387	0.727387	0.363694	91
C	Feed rate	2	0.041749	0.041749	0.020874	5
D	Depth of cut	2	0.004404	0.004404	0.002202	1
	Total	17	0.797616			

### Optimization of GRA and PSI for optimum response parameters

**Fig. 9:**
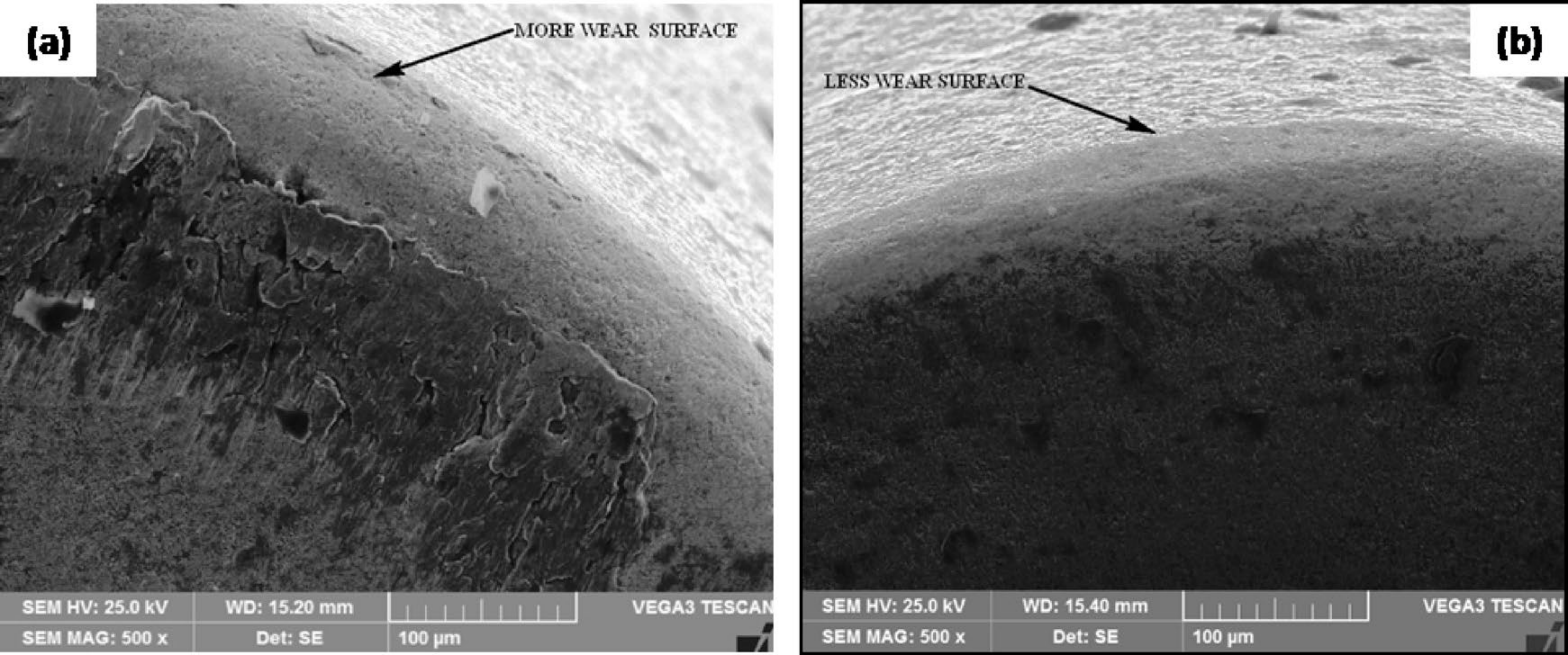
SEM images of (**a**) UT tool (**b**) DCT tool.

**Fig. 10:**
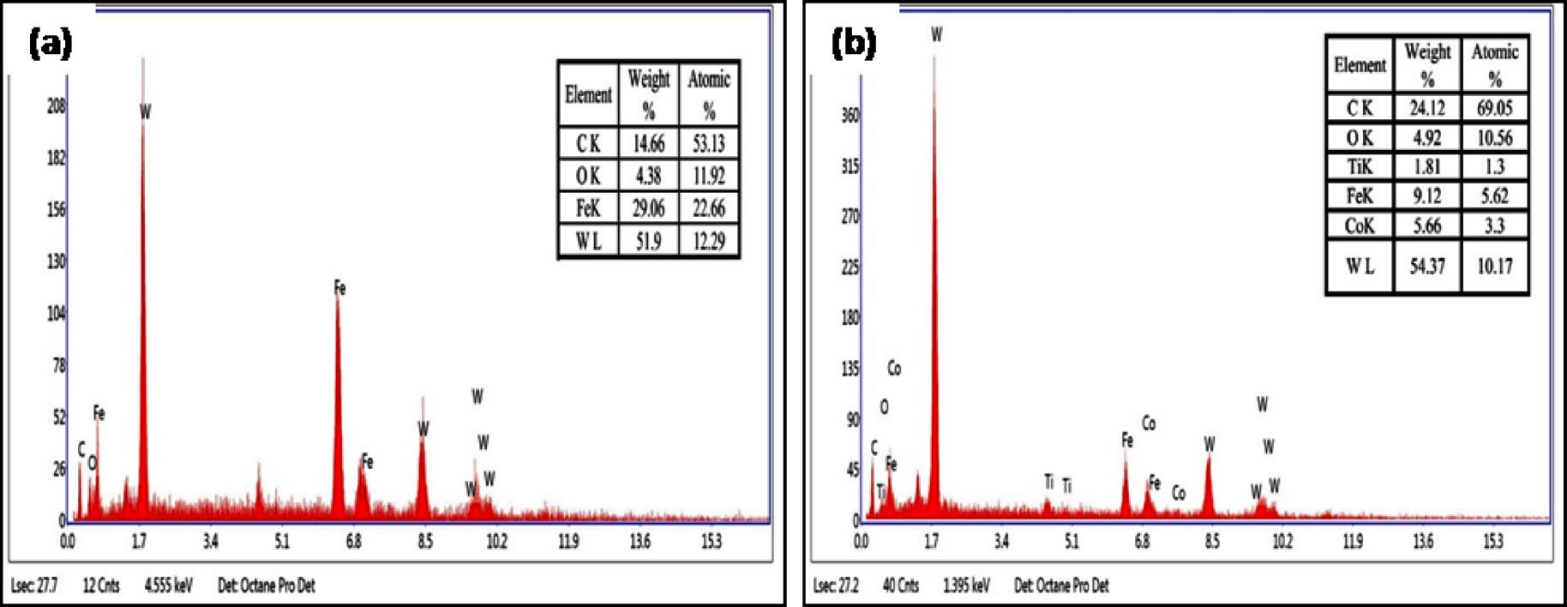
EDX spectra of (**a**) UT tool (**b**) DCT tool.

**Fig. 11:**
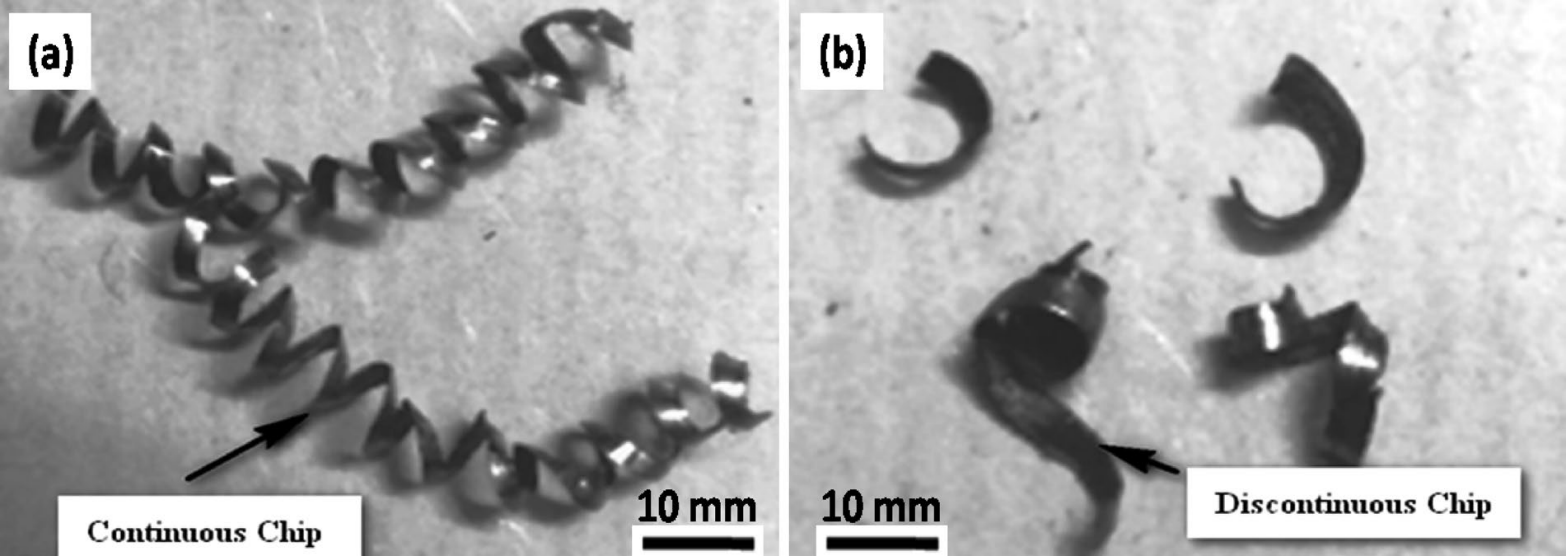
Chips of (**a**) UT tool (**b**) DCT tool.

Based on the parameters of both optimization methods, ANOVA showed that the feed rate, cutting speed, treated tool, and depth of cut all seemed to have an effect on the results, each contributing 55%, 24%, 8%, and 5%, respectively. The contribution was a major effect on the cutting speed and feed rate of the experiment, with the tool and depth of cut, respectively, following at 91%, 5%, 1%, and 1%. When are eliminate the feed rate and cutting speed during machining the most significant factors with other parameters following in order of importance. This has a direct impact on the physical assistance afforded by the adhesion and shear. The cutting speed and input significantly influence tool attrition and failure^30^. Therefore, Figs. 9a-b demonstrates that the DCT tool achieves a less worn surface than the UT tool, as evidenced by the SEM image. A conducted EDAX analysis at the tool rake face of both inserts to evaluate the diffusion at the tool-workpiece interface. Figures 10 (a) and (b) depict the EDAX spectra and chemical compositions of both inserts. The chip’s continuous embossment over the rake face for an extended period of time led to adhesion. This behavior is the result of the reactive integrity of tungsten (W), carbon (C), and iron (Fe). The CT contains a significantly lower quantity of Fe (9.12%) than the UT (29.06%), as indicated in the EDAX analysis. Furthermore, the carbon content suggested that the incidence of DCT was higher (24.12%) than that of UT (14.66%). The adhesive degradation was more intense in the UT inserts, as indicated by the EDAX analysis. The case of built-up edge formation in the UT tool was evident in the substantial amount of chemical composition of the workpiece material that was discovered over the rake face. Conversely, the DCT tool exhibited a negligible quantity of workpiece material. Consequently, the UT tool experienced the most wear due to adhesive wear and uncontrolled chip removal, while the DCT tool exhibited less wear due to the decreased feed rate of built-up edge development over the rake face, as illustrated in Fig. 11a and b^12,31–34^.

## Conclusions

The turning was conducted on an AISI 1045 tungsten carbide tool under UT and DCT conditions, with a variety of cutting speeds, feed rates, and depth of cuts. The impact of a DCT tool on surface roughness and flank wear was investigated using a UT tool. The experimental results led to the following conclusions.

1. The grey relation analysis revealed that the experimental run had the best multi response among the 16 runs with the best grey relation grade. The optimum values for these parameters are A2, B3, C1, and D1. A deep cryo-treated tool (A2) with a cutting speed (B3) of 120 m/min and feed rate (C1) of 0.050 mm/rev. and a depth of cut (D1) of 0.10 mm were the best parameters. With contributions of 55%, 24%, 8%, and 5%, the ANOVA showed that the feed rate, cutting speed, deep cryo tool, and depth of cut had the largest influence on the results.
2. The preference selection index technique revealed that the experimental run had the best multi-response among the 16 runs with the best preference selection index rank Parameters A2, B3, C1, and D3 were the most desirable values. A deep cryo-treated tool(A2), cutting speed (B3) of 120 m/min, feed rate (C1) of 0.050 mm/rev, and depth of cut(D3) of 1.000 mm were the best parameters. ANOVA revealed that cutting speed and feed rate, followed by the deep cryo tool and depth of cut, seemed to influence the results, with contributions of 91%, 5%, 1%, and 1%, respectively.
3. Cryo-treated uncoated tungsten carbide tools have a positive effect on flank wear and surface roughness. The results showed a 17% reduction in flank wear and a 7% reduction in surface finish.

The proposed method revealed that the cutting speed, feed rate, tool, and depth of cut were the parameters that influenced the process selection. The findings revealed that deep cryo-treatment can improve performance, leading to a cost-effective overall machining process. Consequently, they produce a diverse array of steels suitable for industrial applications. The turning performance of the treated tool is enhanced through the utilization of grey relational analysis and preference selection index. This method is both feasible and effective in the creation of material that is adaptable, flexible, and durable.

## Data Availability

All data generated or analyzed during this study are included in this published article.
